# Honokiol abrogates leptin-induced tumor progression by inhibiting Wnt1-MTA1-β-catenin signaling axis in a microRNA-34a dependent manner

**DOI:** 10.18632/oncotarget.3844

**Published:** 2015-04-15

**Authors:** Dimiter B. Avtanski, Arumugam Nagalingam, Panjamurthy Kuppusamy, Michael Y. Bonner, Jack L. Arbiser, Neeraj K. Saxena, Dipali Sharma

**Affiliations:** ^1^ Department of Oncology, Johns Hopkins University School of Medicine and The Sidney Kimmel Comprehensive Cancer Center at Johns Hopkins, Baltimore, MD, USA; ^2^ Department of Medicine, University of Maryland School of Medicine, Baltimore, MD, USA; ^3^ Department of Dermatology, Emory University School of Medicine, Winship Cancer Institute, Atlanta, GA, USA; ^4^ Atlanta Veterans Administration Medical Center, Atlanta, GA, USA

**Keywords:** honokiol, leptin, miR-34a, breast cancer

## Abstract

Obesity greatly influences risk, progression and prognosis of breast cancer. As molecular effects of obesity are largely mediated by adipocytokine leptin, finding effective novel strategies to antagonize neoplastic effects of leptin is desirable to disrupt obesity-cancer axis. Present study is designed to test the efficacy of honokiol (HNK), a bioactive polyphenol from *Magnolia grandiflora*, against oncogenic actions of leptin and systematically elucidate the underlying mechanisms. Our results show that HNK significantly inhibits leptin-induced breast-cancer cell-growth, invasion, migration and leptin-induced breast-tumor-xenograft growth. Using a phospho-kinase screening array, we discover that HNK inhibits phosphorylation and activation of key molecules of leptin-signaling-network. Specifically, HNK inhibits leptin-induced Wnt1-MTA1-β-catenin signaling *in vitro* and *in vivo.* Finally, an integral role of miR-34a in HNK-mediated inhibition of Wnt1-MTA1-β-catenin axis was discovered. HNK inhibits Stat3 phosphorylation, abrogates its recruitment to miR-34a promoter and this release of repressor-Stat3 results in miR-34a activation leading to Wnt1-MTA1-β-catenin inhibition. Accordingly, HNK treatment inhibited breast tumor growth in diet-induced-obese mouse model (exhibiting high leptin levels) in a manner associated with activation of miR-34a and inhibition of MTA1-β-catenin. These data provide first *in vitro* and *in vivo* evidence for the leptin-antagonist potential of HNK revealing a crosstalk between HNK and miR34a and Wnt1-MTA1-β-catenin axis.

## INTRODUCTION

Obesity has emerged as a global health problem with prevalence rates dramatically increasing in the last two decades in the United States and many other countries. According to WHO criterion for classifying obesity [body mass index (BMI) <18.5 (underweight); 18.5-24.9 (normal); 25.0-29.9 (overweight); ≥; 30.0 kg/m^2^ (obese)], an estimated 36% of American adults are obese and 33% are overweight. Worldwide approximately 500 million adults are obese and 1.1 billion are overweight [1, 2]. Multitudes of epidemiological studies have shown that obesity greatly influences risk, progression and prognosis of various cancers including breast cancer [3, 4]. Results from the Million Women study examining breast cancer incidence and mortality in relation to obese state show that approximately half of the cancers in postmenopausal women can be attributed to high BMI [5]. Advanced grade and stage including lymph node metastasis are more prevalent in obese women with invasive breast cancer which partly explains why obese women in the highest quintile of BMI have double the death rate from breast cancer when compared with women in the lowest quintile [6, 7]. In addition to being associated with postmenopausal estrogen receptor (ER)-positive breast cancer, obese state is also associated with ER-negative breast cancer with high S-phase fraction, histological grade, mitotic cell count, expression levels of proliferation markers, and a larger tumor size [8].

Hypertrophy and hyperplasia of adipocytes, typically associated with obese state, greatly alters the local and systemic secretion of biologically active polypeptides, adipocytokines such as leptin. Acting by endocrine, paracrine, and autocrine mechanisms, dysregulated leptin affect various oncogenic processes [9, 10]. High level of plasma leptin is linked with increased risk and poor prognosis for breast carcinogenesis in epidemiological studies [11, 12]. We and others have established that high leptin levels (hyperleptinemia) associated with obese state stimulate breast cancer cell proliferation, invasion, migration, and angiogenesis, thereby promoting breast tumor growth and metastasis [13-19]. Given that leptin and leptin-signaling pathway are prime targets for disrupting obesity-breast cancer link, developing effective, non-endocrine, non-toxic agents for prevention of the neoplastic effects of leptin are highly important.

The importance of active constitutive agents in natural products has become increasingly apparent owing to their potential cancer preventive as well as therapeutic properties [20, 21]. Traditional asian medicine has successfully used cones, bark and leaves from Magnolia plant species for their anti-thrombocytic, anti-inflammatory, anxiolytic, anti-depressant, antioxidant, antispasmodic, antibacterial and anticancer effects [22-25]. Medicinal benefits of *Magnolia* species have been assigned to honokiol (HNK), a natural phenolic compound isolated from an extract of seed cones from *Magnolia grandiflora* [26]. Previous studies from our lab have shown that HNK inhibits breast carcinogenesis *in vitro* and *in vivo* [27, 28]. In the present study, we specifically investigated the potential of HNK to inhibit oncogenic effects of highly-active leptin signaling pertaining to obese state and examine the underlying molecular mechanisms. Our *in vitro* and *in vivo* analyses show that HNK inhibits breast tumorigenesis in obese state, inhibits leptin-induced Wnt1-MTA1-β-catenin signaling via miR-34a activation in a Stat3-dependent manner.

## RESULTS

### Honokiol impedes leptin-induced clonogenicity, anchorage-independent growth, invasion, and migration of breast cancer cells and inhibits breast tumor progression in athymic nude mice treated with leptin

**Figure 1:**
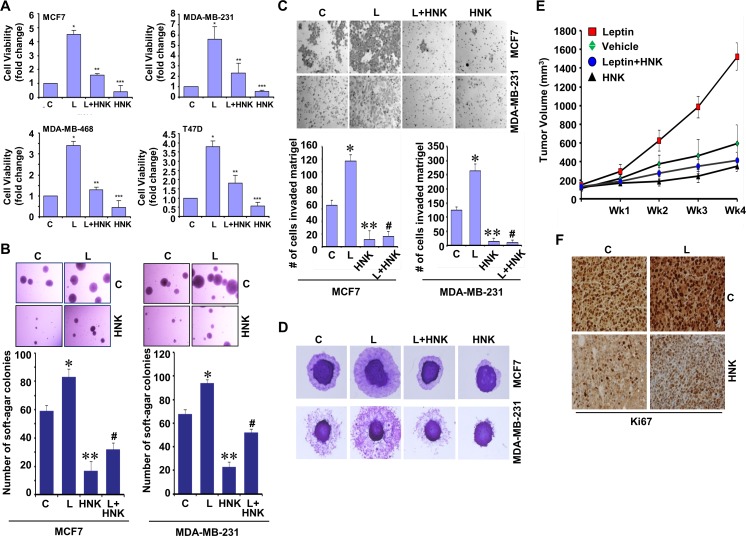
Honokiol diminishes the stimulatory effect of leptin on cell viability, anchorage-independent growth, invasion, migration and breast tumor growth in nude mice **A**. Breast cancer cells were treated with leptin and/or HNK as indicated and cell viability was examined by trypan blue dye exclusion assay. *p < 0.05 compared with untreated controls. Vehicle-treated cells are denoted with C. **B**. Soft-agar colony-formation of breast cancer cells treated with HNK and/or L as in A for three weeks. Histogram represents average number of colonies counted (in six micro-fields). *, *P* < 0.001, compared with Vehicle-treated cells (C); **, *P* < 0.005, compared with controls; #, *P* < 0.001, compared to leptin-treated cells. **C**. Analysis of Matrigel invasion of breast cancer cells treated as in A. Representative images are shown. The histogram shows mean of three independent experiments performed in triplicates. *, *P* < 0.005, compared with vehicle-treated controls (C); **, *P* < 0.001, compared with controls; #, *P* < 0.001, compared to leptin-treated cells. **D**. Spheroid migration assay of breast cancer cells in the presence of 100 ng/ml leptin (L), 5 μM HNK alone and in combination. The spheroids were photographed at 48h-post treatment. The results shown are representative of three independent experiments performed in triplicates. **E**. MDA-MB-231 cells derived tumors were developed in nude mice and treated with vehicle, Leptin, Honokiol (HNK) or Leptin + HNK. Tumor growth was monitored by measuring the tumor volume for 4 weeks. (*n* = 8-10); (*P* < 0.001). **F**. Tumors from vehicle (C), Leptin (L), HNK or HNK+L-treated mice were subjected to immunohistochemical (IHC) analysis using Ki-67 antibodies.

Multiple epidemiological, clinical and preclinical studies have shown the importance of adipocytokine leptin in mediating the molecular effects of obesity [11, 12]. Recent studies from our lab and others have revealed myriad oncogenic effects of leptin including induction of growth, epithelial-mesenchymal-transition (EMT), invasion and migration potential of breast cancer [13-19]. Here, we specifically examined if HNK could inhibit the oncogenic effects of leptin on breast cancer growth and metastatic properties using well-characterized human breast cancer cell lines (MCF-7, MDA-MB-231, MDA-MB-468 and T47D) as models. We first examined the effect of HNK on leptin-induced cell-viability, clonogenic potential and anchorage-independent growth of breast cancer cells. Treatment with 5 μM HNK resulted in significant (50-60%) inhibition of leptin-induced cell-viability, clonogenicity and soft-agar colony-formation (Figure 1A, 1B and Supplementary Figure 1). Next, we investigated the efficacy of HNK to block leptin-induced invasion and migration of breast cancer cells using Matrigel invasion and spheroid migration assay. Breast cancer cells exhibited augmentation of invasion and migration potential upon leptin treatment which was effectively inhibited with HNK treatment (Figure 1C, 1D). We further investigated the *in vivo* physiological relevance of our *in vitro* findings by evaluating whether HNK treatment had inhibitory effects on the development of breast carcinoma in leptin-treated nude mouse models. As evident in Figure 1E, breast tumor growth was significantly accelerated in leptin-treated experimental group in comparison to the control group. Displaying noteworthy *in vivo* efficacy against oncogenic effects of leptin, HNK treatment reduced breast tumor growth in leptin + HNK treated experimental group (Figure 1E). Ki-67, a nuclear non-histone protein, is one of the major markers of tumor proliferation [29] used as a decision-making tool for adjuvant therapy [30]. The immunohistochemical assessment of tumor proliferation showed higher Ki-67 in the leptin-treated group as compared with the control group. Co-treatment with HNK and leptin exhibited reduced levels of Ki-67 in comparison to leptin-treated group (Figure 1F). Together, these results demonstrate that HNK treatment results in effective suppression of leptin-induced breast tumor growth suggesting that HNK is a novel and effective leptin-antagonist.

### Honokiol mediated alterations in phosphorylation of key leptin-signaling mediators in breast cancer cells

**Figure 2:**
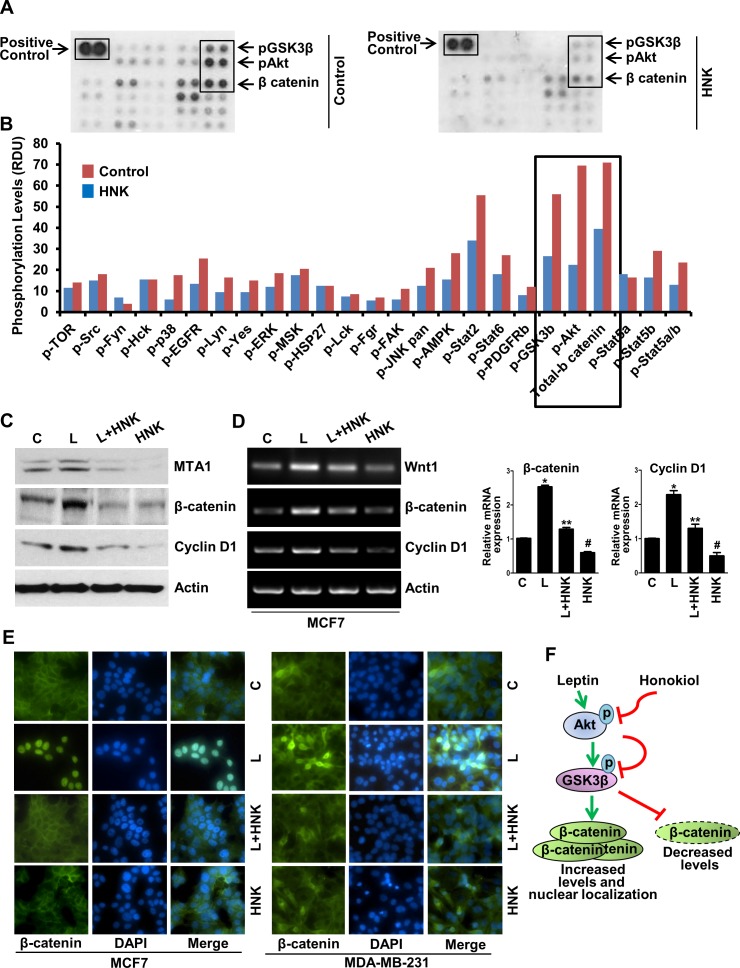
Human phospho-antibody array analyses reveal HNK-induced decreased phosphorylation of key leptin-signaling components and HNK decreases leptin-induced expression and nuclear translocation of β-catenin **A, B**. MCF7 cells were treated with 5 μM HNK for 6 hours and subjected to Human phospho-antibody array analyses. Relative levels of protein phosphorylation (normalized intensity for each antibody) were calculated for each untreated and treated sample. *, *P* < 0.001, compared with untreated controls. **C**. Immunoblot analysis and **D**. RT-PCR and real-time analysis of MTA1, β-catenin and cyclin D1 in breast cancer cells treated with vehicle (C), 5 μM HNK and 100ng/ml leptin (L) alone and in combination as indicated. **E**. Breast cancer cells were treated with 5 μM HNK and 100ng/ml leptin alone and in combination as indicated and subjected to immunofluorescence analysis of β-catenin. Nuclei were visualized with DAPI staining. Vehicle-treated cells are denoted with ‘C’. **F**. Schematic representation.

Activation or inhibition of specific kinases via phosphorylation/dephosphorylation plays an important role in mediating the biological effects of extracellular stimuli, enabling them to control multiple signal-transduction pathways and ultimately, cellular functions. To identify signaling networks involved in HNK-induced inhibition of breast carcinogenesis, we interrogated 46 specific Ser/Thr/Tyr phosphorylation sites of 38 selected proteins using phosphoprotein arrays. Breast cancer cells were treated with 5 μM HNK for 6h and subjected to phospho-protein analysis. We discovered that phosphorylation of protein kinase B (Akt-S473) and glycogen synthase kinase-3β (GSK3β-S21/S9) was significantly decreased in breast cancer cells treated with HNK. In addition, expression level of total β-catenin was also considerably reduced upon HNK treatment (Figure 2A, 2B, and Supplementary Figure 2). Previous studies from our lab have implicated Akt-GSK3β and β-catenin in oncogenic function of leptin [15, 31, 32]. We show that leptin transmits signals via both MTA1 (metastasis associated antigen)-Wnt1 and Akt pathways that cause the phosphorylation, hence inactivation of GSK3β leading to dissociation of GSK3β-Axin destruction complex. Inactivation of destruction complex allows accumulation and nuclear translocation of β-catenin, alteration of responsive gene expression and ultimately induces growth and metastatic progression of breast tumors in response to leptin treatment [15]. Our finding that HNK modulates Akt, GSK3β, and β-catenin, key components of leptin-signaling network in breast cancer cells, prompted us to further explore whether HNK treatment can also inhibit MTA1, Wnt1, β-catenin and cyclin D1 in the presence of leptin. Treatment of breast cancer cells with leptin exhibited striking increase in MTA1, Wnt1, β-catenin and cyclin D1 expression in comparison to untreated cells. HNK treatment efficiently inhibited leptin-induced expression of MTA1, Wnt1, β-catenin and cyclin D1 (Figure 2C, 2D and Supplementary Figure 3). Given our previous findings that nuclear translocation of β-catenin is integral to leptin-induced gene activation [15], we investigated the effect of HNK on leptin-induced nuclear translocation of β-catenin. Treatment of MCF7 and MDA-MB-231 cells with leptin showed nuclear accumulation of β-catenin and co-treatment with HNK inhibited leptin-induced expression and nuclear translocation of β-catenin (Figure 2E and Supplementary Figure 4). Figure 2F shows a schematic representation of leptin-induced β-catenin pathway and the effect of HNK on this pathway.

### Honokiol administration abrogates leptin-induced MTA1-Wnt1-β-catenin-cyclin D1 axis *in vivo* and inhibits breast tumor progression in obese hyperleptinemic state

**Figure 3:**
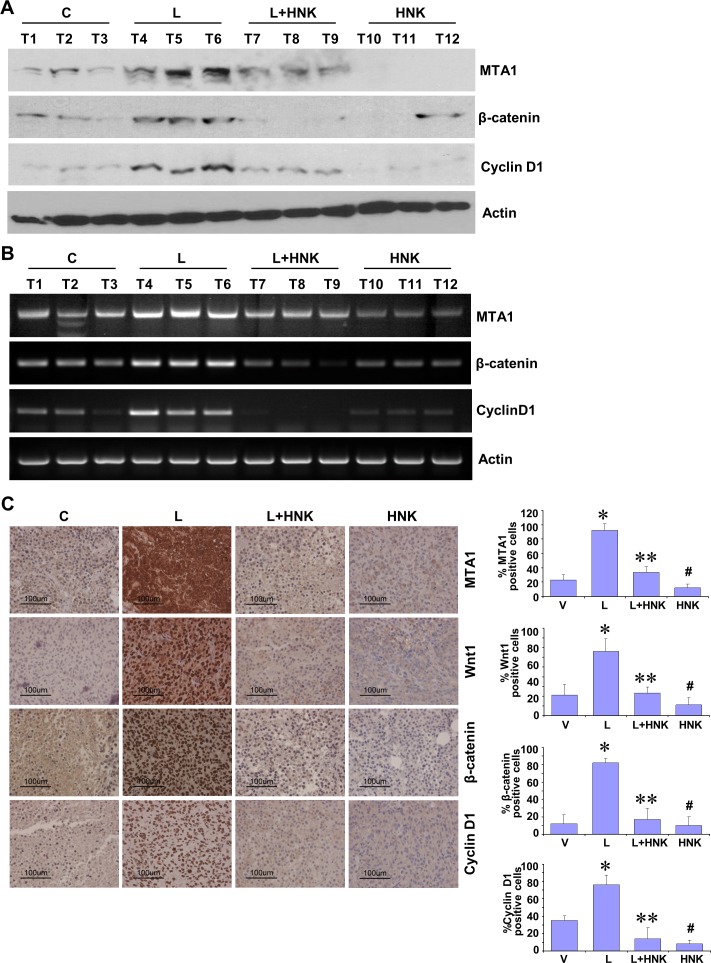
*In vivo* evidence for HNK-mediated inhibition of leptin-induced MTA1/Wnt1/β-catenin pathway in breast cancer cells MDA-MB-231 cells derived tumors were developed in nude mice and treated with Leptin (L), HNK, Leptin + HNK or vehicle (C). At the end of four weeks of treatment, tumors were collected for analysis. **A.** Total protein lysates from breast tumor samples were immunoblotted for MTA1, β-catenin, cyclin D1 expression levels. Actin was used as control. **B.** Total RNA was isolated from tumor samples and subjected to RT-PCR analysis. Expression of MTA1, β-catenin, cyclin D1 was analyzed. Actin was used as control. **C.** Breast tumors from each treatment group were subjected to immunohistochemical analysis using MTA1, Wnt1, β-catenin and cyclin D1 antibodies. Bar diagram shows quantitation of MTA1, Wnt1, β-catenin and cyclin D1 expression in tumors from each treatment group. Columns, mean (*n* = 5); *, *P* < 0.005, compared with control; **, *P* < 0.001, compared with leptin-treatment; #, *P* < 0.05, compared to untreated cells.

**Figure 4:**
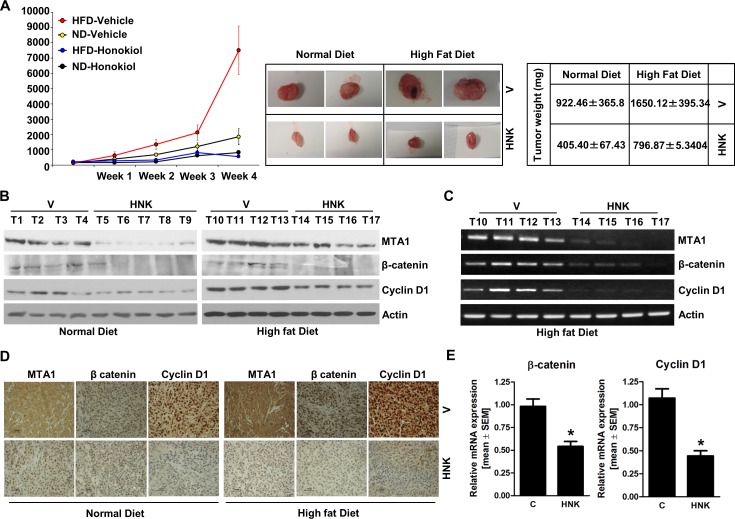
HNK treatment inhibits breast tumor growth in obese state, and inhibits MTA1-β-catenin, cyclin D1 axis MDA-MB-231 cells derived tumors were developed in high-fat-diet (HFD) fed obese mice and normal-diet (ND) fed non-obese mice and treated with oral HNK or vehicle (V). At the end of treatment, tumors were collected for analysis. **A.** Tumor growth was monitored by measuring the tumor volume for 4 weeks. (*n* = 8-10); (*P* < 0.001). **B.** Total protein lysates from breast tumor samples were immunoblotted for MTA1, β-catenin, cyclin D1 expression levels. Actin was used as control. **C.** Total RNA was isolated from tumor samples and subjected to RT-PCR analysis **C.** and real-time PCR analysis **E.**. Expression of MTA1, β-catenin, cyclin D1 was analyzed. Actin was used as control. **D.** Breast tumors from each treatment group were subjected to immunohistochemical analysis using MTA1, β-catenin and cyclin D1 antibodies.

Our studies show that HNK treatment inhibits leptin-induced breast tumor progression *in vivo* (Figure 1E). We utilized tumor tissue samples from the same experiment to examine the effect of HNK treatment on the expression and activation of important leptin-signaling molecules. Tumors from the leptin-treated mice exhibited increased expression of MTA1, β-catenin and cyclin D1 in comparison to the vehicle-treated group. HNK treatment resulted in effective inhibition of leptin-induced MTA1, β-catenin and cyclin D1 (Figure 3A). Also, leptin treated tumors showed transcriptional upregulation of MTA1, β-catenin and cyclin D1 while HNK treatment inhibited their leptin-induced expression (Figure 3B). Immunohistochemical analysis showed that tumors from leptin-treated mice exhibited higher number of tumor cells showing increased expression of MTA1, Wnt1, β-catenin and cyclin D1 as compared to tumors from vehicle-treated group. Tumors from HNK+leptin treatment group showed very low percentage of tumor cells showing expression of MTA1, Wnt1, β-catenin and cyclin D1 providing physiological relevance to our *in vitro* findings (Figure 3C). To further investigate the efficacy of HNK treatment on breast tumor progression in hyperleptinemic obese state, we utilized diet-induced obesity model. High-fat diet fed (HFD) mice exhibited a significant increase in leptin level (ND = 6.1 ± 0.6 ng/ml *vs*. HFD = 35± 4.3 ng/ml) and increased body weight (ND = 18 ± 1.5 g *vs*. HFD = 34± 2.2 g) in comparison to normal-diet (ND) group (Supplementary Figures 5 and 6). Oral HNK administration significantly inhibited the accelerated breast tumor growth observed in HFD group (Figure 4A). Next, we examined the effect of HNK on the expression of important leptin-signaling molecules. Breast tumors from HFD group exhibited elevated expression of MTA1, β-catenin and cyclin D1 which was efficiently abrogated by HNK treatment (Figure 4B). HNK treatment also inhibited transcriptional upregulation of MTA1, β-catenin and cyclin D1 in HFD tumors (Figure 4C, 4E). Tumors from HFD and ND groups treated with vehicle and HNK were subjected to immunohistochemical analysis. Higher percentage of tumor cells showed increased expression of MTA1, β-catenin and cyclin D1 in HFD group as compared to ND group. It is important to note that HNK treatment inhibited the expression of MTA1, β-catenin and cyclin D1 in ND group as well as HFD group (Figure 4D). Collectively, the findings presented here show that HNK treatment inhibits tumor progression in hyperleptinemic (high-leptin) obese conditions and abrogate activation of leptin-signaling molecules.

### Inhibition of Wnt1-β-catenin axis contributes to HNK-mediated inhibition of breast cancer growth

**Figure 5:**
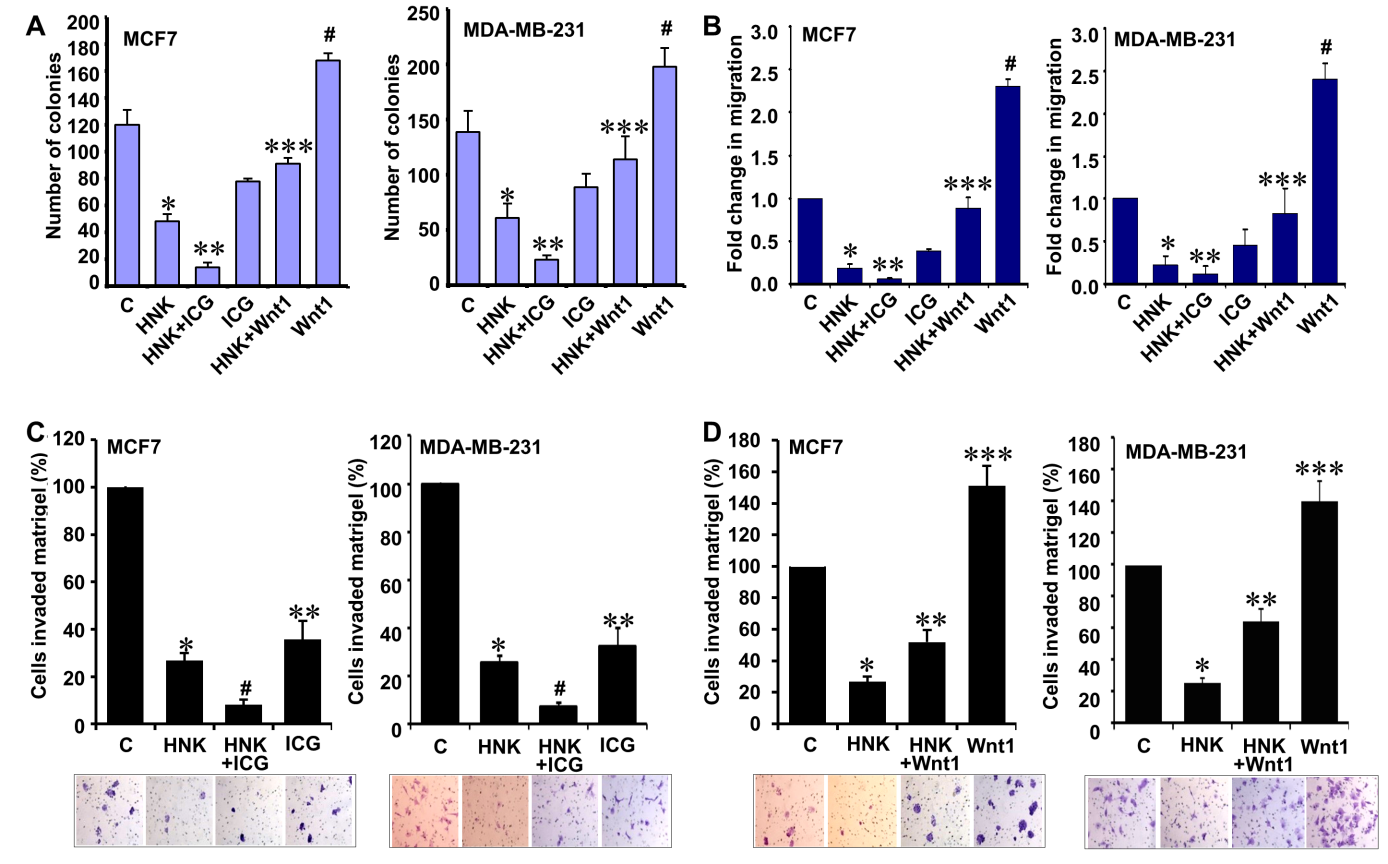
Inhibition of β-catenin potentiates while Wnt1 treatment reduces the effect of HNK on invasion and migration potential of breast cancer cells **A.** Clonogenicity of breast cancer cells treated with 5 μM HNK, ICG001 or Wnt1 alone and in combination as indicated. Vehicle-treated cells are denoted with the letter “C”. *, *P* < 0.001, compared with untreated controls; **, *P* < 0.005, compared to HNK-treated cells; ***, *P* < 0.001, compared with HNK-treated controls; #, *P* < 0.005, compared to untreated cells. **B.** MCF7 and MDA-MB-231 cells were subjected to spheroid-migration assay. Culture media were replaced with media containing 5 μM HNK, ICG001, Wnt1 alone and in combination as indicated. The spheroids were photographed at 48h-post treatment. The results (fold-change in migration) shown are representative of three independent experiments performed in triplicates. *, *P* < 0.005, compared with untreated controls; **, *P* < 0.005, compared to HNK-treated cells; ***, *P* < 0.001, compared with HNK-treated controls; #, *P* < 0.001, compared to untreated cells. **C.** Matrigel invasion assay of breast cancer cells treated with 5 μM HNK and ICG001 alone and in combination as indicated for 24 hours. The number of cells that invaded through the Matrigel was counted in five different regions. The slides were blinded to remove counting bias. Representative images are shown. The results show mean of three independent experiments performed in triplicates. *, *P* < 0.005, compared with untreated controls; #, *P* < 0.001, compared to HNK-treated cells; **, *P* < 0.001, compared with untreated controls. **D.** Matrigel invasion assay of breast cancer cells treated with 5 μM HNK and Wnt1 alone and in combination as indicated for 24 hours. Representative images are shown. The results show mean of three independent experiments performed in triplicates. *, *P* < 0.001, compared with untreated controls; **, *P* < 0.001, compared to HNK-treated cells; ***, *P* < 0.001, compared with untreated controls. Vehicle-treated cells are denoted with the letter “C”.

Wnt1 expression is upregulated in various cancers including breast cancer [33]. We previously show that leptin induces canonical activation of Wnt1 signaling through β-catenin-dependent mechanisms in breast cancer cells [15]. Given our *in vitro* and *in vivo* results showing that HNK inhibits Wnt1-β-catenin axis and its important role in oncogenic function of leptin, we decided to examine whether inhibition of Wnt1-β-catenin axis is integral for HNK-mediated inhibition of breast cancer growth, invasion and migration. We reasoned that targeted inhibition of this signaling using a small molecule inhibitor might be able to potentiate HNK-mediated growth inhibition whereas treatment with purified Wnt1 might interfere with HNK efficacy. Utilizing a peptidomimetic small molecule (ICG-001) that has been shown to inhibit β-catenin signaling [34, 35], we observed that combined treatment of ICG and HNK resulted in further reduction of growth in comparison to HNK treatment alone. Owing to its oncogenic function [33], Wnt1 treatment resulted in increased growth of breast cancer cells and Wnt1 treatment abrogated HNK-mediated growth inhibition (Figure 5A). As shown in Figure 5B, HNK inhibited migration of breast cancer cells which was additionally reduced with ICG co-treatment while Wnt1 co-treatment interfered with HNK-mediated inhibition of migration. Wnt1 treated cells exhibited increased migration (Figure 5B). Effective inhibition of invasion of breast cancer cells was observed with HNK treatment. Supplementation of HNK with ICG resulted in more effective inhibition of invasion of breast cancer cells (Figure 5C). Wnt1 treatment alone increased invasion of breast cancer cells. Wnt1 abrogated HNK-mediated inhibition of invasion of breast cancer cells (Figure 5D). Based on our studies, we propose a model in which inhibition of Wnt1-β-catenin axis is important for HNK-mediated inhibition of breast cancer growth, invasion and migration.

### HNK inhibits Wnt1-β-catenin axis via upregulation of miR-34a in a Stat3-dependent manner

**Figure 6:**
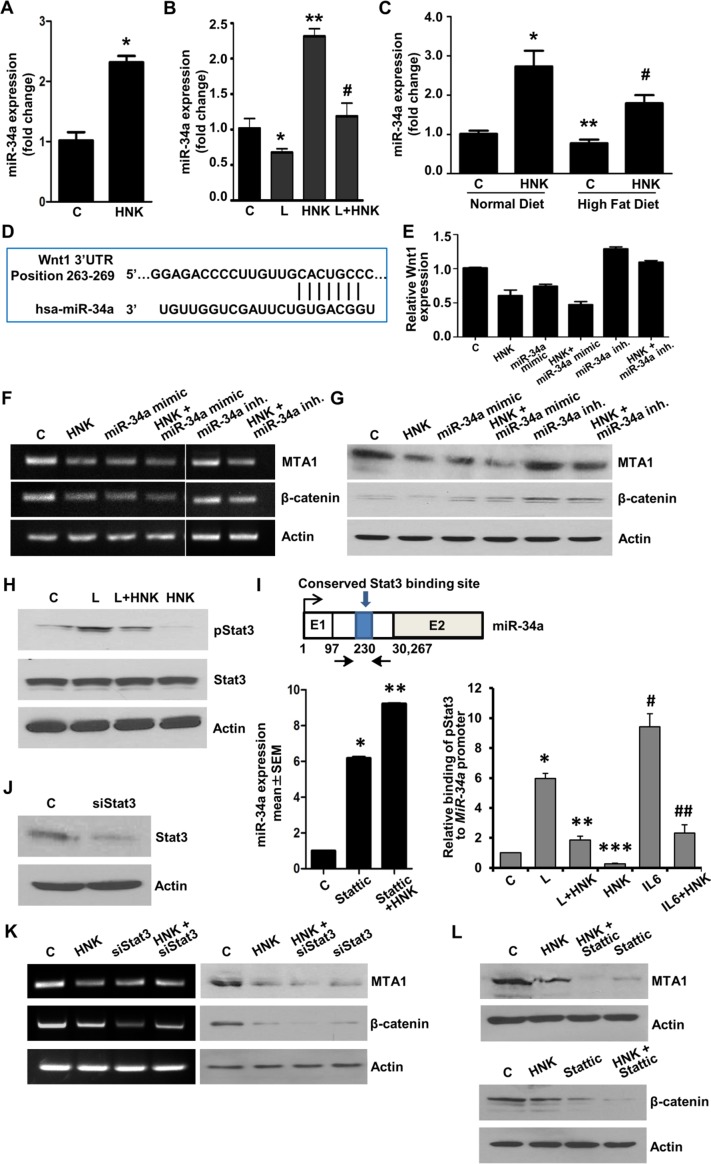
Involvement of miR-34a in HNK-mediated inhibition of MTA1-β-catenin axis and role of Stat3 inhibition in HNK-mediated miR34a upregulation and MTA1-β-catenin axis inhibition **A.** Expression levels of miR-34a in breast cancer cells treated with 5 μM HNK. *, *P* < 0.001, compared with untreated controls **B.** Expression levels of miR-34a in breast cancer cells treated with 100 ng/ml leptin and 5 μM HNK alone and in combination. *, *P* < 0.005, compared with controls; **, *P* < 0.001, compared to controls; #, *P* < 0.005, compared with leptin-treated cells. **C.** Expression levels of miR-34a in tumor samples from high-fat-diet (HFD) fed obese mice group +vehicle, normal-diet (ND) fed non-obese mice group + vehicle, HFD+HNK and ND+HNK is quantified by using TaqMan MicroRNA Assays-based RT-PCR. *, *P* < 0.001, compared with ND controls; **, *P* < 0.005, compared to ND controls; #, *P* < 0.001, compared with HFD controls. **D.** Schematic representation of the predicted miR-34a MREs within the 3′UTR of the Wnt1 mRNA. Alignment between the miR-34a binding site and miR-34a is shown. **E.** Breast cancer cells were transfected with miR-34a inhibitor or miR-34a mimic followed by treatment with vehicle (C) or HNK as indicated. Wnt1 expression was analyzed using real-time PCR analysis. **F, G.** Breast cancer cells were transfected with miR-34a inhibitor or miR-34a mimic followed by treatment with vehicle (C) or HNK as indicated. RT-PCR analysis **F.** and immunoblot analysis **G.** of MTA1, and β-catenin was performed. Actin was used as control. **H.** Immunoblot analysis of phosphorylated-Stat3, and Stat3 in breast cancer cells treated with 5 μM HNK and 100ng/ml leptin alone and in combination as indicated. **I.** Schematic shows map of the human *MIR-34A* genomic region with the indicated conserved Stat3-binding site. Expression levels of miR-34a in breast cancer cells treated with 5 μM HNK and 10 μM Stattic as indicated. *, *P* < 0.005, compared with controls (C); **, *P* < 0.005, compared to Stattic. ChIP analysis of Stat3 recruitment at the human *MIR-34A* in breast cancer cells treated with 100ng/ml leptin (L), 5 μM HNK and IL-6 as indicated. *, *P* < 0.001, compared with controls; **, *P* < 0.001, compared to L; ***, *P* < 0.001, compared to C; #, *P* < 0.001, compared to C; ##, *P* < 0.001, compared to Il-6. **J.** Breast cancer cells were transfected with siStat3 or control miR and expression of Stat3 was analyzed using immunoblotting. **K.** RT-PCR analysis of MTA1 and β-catenin in breast cancer cells transfected with siStat3 and treated with 5 μM HNK as indicated. **L.** Immunoblot analysis of MTA1 and β-catenin in breast cancer cells treated with 5 μM HNK and 10 μM Stattic as indicated.

In recent years, microRNAs (miRs) have been recognized as important regulators of diverse biological processes as individual miRs can repress multiple genes. miRs are small (~21-mer) regulatory RNA molecules that bind to the 3′untranslated regions (3′UTR) of specific mRNAs and trigger mRNA degradation or translational repression [36]. While several miRNAs have been proposed to regulate various aspects of breast cancer growth and metastatic progression [37], miR-34a has emerged as an important tumor suppressor affecting tumor cell apoptosis, proliferation, migration and invasion and, importantly, is downregulated in aggressive breast tumors [38]. We discovered that HNK treatment increases the expression of miR-34a in breast cancer cells (Figure 6A). It is interesting to note that leptin treatment decreased miR-34a expression while HNK increased miR-34a expression. Cells treated with a combination of leptin and HNK showed a mitigated response to HNK (Figure 6B). These findings were validated utilizing breast tumors from our *in vivo* studies. Analysis of breast tumors showed elevated levels of miR-34a in HNK-treated normal-diet (ND) group. Breast tumors from high-fat-diet (HFD)-induced obese hyperleptinemic mice exhibited lower miR-34a expression which could be rescued with HNK treatment (Figure 6C). To further explore the role of miR-34a in HNK function, we searched for potential downstream target genes using (i) bioinformatic algorithms (TargetScan, RNA22, and miRBase); and (ii) analyzing the gene expression profile induced by miR-34a from previous microarray profile studies [39, 40]. Interestingly, Wnt1 ranked high in the list of miR-34a predicted target genes and we found a miR-34a binding site at nucleotides 263-269 of the Wnt1 3′UTR (Figure 6D). We further examined the role of miR-34a in HNK-mediated inhibition of Wnt1-β-catenin axis. Ectopic miR-34a expression (in the form of a mimic molecule) further enhanced HNK-mediated inhibition of Wnt1-MTA1-β-catenin axis while expression of miR-34a-inhibitor abrogated the effect of HNK on Wnt1-MTA1-β-catenin axis (Figure 6E, 6F, 6G). These studies presented miR-34a as an important node in HNK-mediated inhibition of Wnt1-MTA1-β-catenin axis in breast cancer cells.

**Figure 7:**
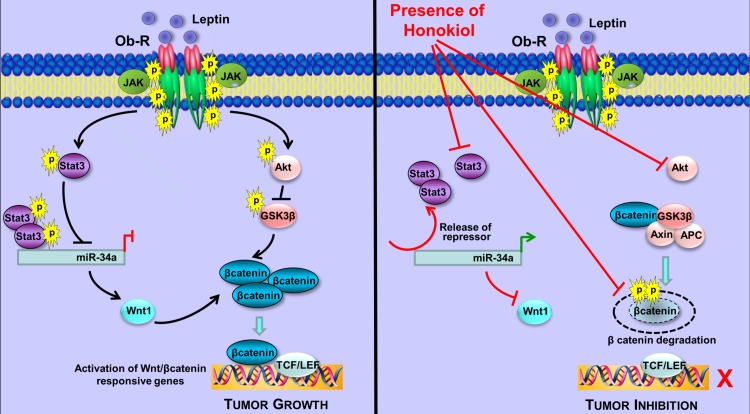
Schematic representation of the crosstalk between HNK and Stat3-miR-34a and Wnt1-MTA1-β-catenin axes Leptin treatment induces phosphorylation of Stat3 leading to increased recruitment of Stat3 to miR34a resulting in repression of miR34a. Inhibition of miR34a allows increased levels of Wnt1 expression in the presence of leptin. Leptin treatment results in stabilization and nuclear localization of βcatenin increasing activation of Wnt/βcatenin responsive genes. Honokiol treatment inhibits leptin-induced phosphorylation of Stat3, releases Stat3 from the conserved Stat3-binding site located in the first intron of miR-34a hence releasing the repressive effects of Stat3 and increasing miR34a expression. miR34a represses Wnt1 expression. Honokiol treatment results in abrogation of Wnt/βcatenin signaling pathway even in the presence of leptin.

Our previous studies have put forth Stat3 activation as an integral event in leptin signaling mediating oncogenic actions of leptin in breast cancer [14]. As evident in Figure 6H, leptin increased Stat3 phosphorylation while HNK treatment effectively inhibited leptin-induced Stat3 phosphorylation. We also showed that high leptin levels (*in vitro* and *in vivo*) resulted in reduced expression of miR-34a (Figure 6B, 6C). A recent study reported a connection between Stat3 and miR-34a where Stat3 directly repressed miR-34a expression [41]. Analysis of miR-34a genomic region showed a phylogenetically conserved Stat3-binding site located in the first intron in close proximity to the first exon (Figure 6I). Accordingly, treatment of breast cancer cells with Stattic (a small-molecule inhibitor of Stat3) inhibited phosphorylation of Stat3 and increased expression of miR-34a, which was further increased with co-treatment with HNK (Figure 6I). Chromatin immunoprecipitation analysis revealed that breast cancer cells treated with leptin and IL-6 (used as a positive control) showed increased recruitment of Stat3 at the miR-34a promoter while HNK treated cells showed decreased Stat3 recruitment. HNK treatment effectively reduced Stat3 binding to miR-34a promoter even in the presence of leptin (Figure 6I). Inhibition of Stat3 via si-Stat3 (Figure 6J) or Stattic treatment also potentiated HNK-mediated inhibition of MTA1 and β-catenin expression in breast cancer cells (Figure 6K, 6L). These results showed the mechanistic insight underlying miR-34a regulation by HNK. HNK inhibits Stat3 phosphorylation, abrogates its recruitment to miR-34a promoter and this release of repressor-Stat3 results in miR-34a activation. Collectively, the findings presented here suggest that HNK inhibits leptin-induced tumor progression and provide *in vitro* as well as *in vivo* evidence for the involvement of miR-34a as a regulator of HNK-mediated inhibition of Wnt1-MTA1-β-catenin axis, and uncover a novel mechanism of HNK action through inhibition of Stat3 leading to miR-34a activation (Figure 7).

## DISCUSSION

Exhibiting multiple molecular changes capable of altering not only the tumor cell characteristics but also various aspects of tumor microenvironment, obesity is a complex physiologic state. Obese state results in up to 2.12-fold increase in the relative risk of cancer-related mortality [42]. Considering the pandemic nature of obesity (approximately two-thirds of US adults are overweight or obese), a 2.12-fold increase in relative risk of cancer-related mortality is a very significant medical problem. Obesity-related changes include hypertrophy and hyperplasia of adipocytes leading to adipocytokine dysregulation manifested by high levels of leptin in obese state. Leptin has been identified as a key member of the obesity-breast cancer molecular network influencing several aspects of breast cancer initiation, growth, metastatic progression as well as response to therapy [13-19]. Developing novel strategies to block specific biologic mechanism(s) by which leptin contributes to breast cancer progression and prognosis is essential for individualizing care for improving outcomes among obese breast cancer patients.

It is recognized that an ideal strategy to inhibit oncogenic effects of leptin should be safe, highly efficacious, and lacks toxicity through long-term use. Leptin antagonism using pharmacologic agents, such as soluble LRs (leptin receptors), synthetic leptin-antagonists, and anti-LR monoclonal antibodies (anti-LR mAbs) [19], although shown to work in certain conditions, are not generally accepted. Given the importance of leptin in driving obesity-breast cancer axis and the current lack of effective, clinically viable leptin-antagonists, it is imperative to develop novel leptin-antagonists that can potentially be utilized for clinical use. The importance of active constitutive agents in natural products has become increasingly apparent owing to their potential cancer preventive as well as therapeutic properties [20, 21]. Our findings showing the ability of bioactive small-molecule agent Honokiol (HNK) to effectively inhibit breast carcinogenesis in a non-toxic, non-endocrine manner [27, 28] render this agent of potential interest in the treatment of breast cancer and spur our interest in further investigating whether HNK is capable of inhibiting breast cancer in obese hyperleptinemic state.

We show that HNK effectively inhibits leptin-induced growth of breast cancer cells *in vitro* and *in vivo.* Importantly, oral HNK treatment also efficiently abrogates breast tumor growth in HFD-induced obese-hyperleptinemic mice. These findings provide strong evidence supporting the efficacy of HNK as a novel leptin-antagonist warranting further mechanistic investigations. Therefore, studies are designed to decipher the key nodes of leptin-antagonist function of HNK to facilitate establishing surrogate biomarkers for its efficacy and help in clinical development of this bioactive molecule as a leptin-antagonist. Phosphokinase array studies lead us to novel discoveries that HNK specifically inhibits the kinase pathway previously revealed by our group for its integral role in leptin function. Indeed, we recently presented *in vitro* and *in vivo* evidence showing that Wnt1-MTA1-β-catenin pathway plays an important role in mediating oncogenic actions of leptin [15]. HNK inhibits leptin-induced Wnt1, MTA1 and β-catenin expression in breast cancer cells. These studies provide an interesting mechanism by which HNK-mediated inhibition of Stat3, in a concerted action, results in concomitant upregulation/activation of miR-34a which is turn results in inhibition of Wnt1. We also identify that Wnt1/β-catenin inhibition is important for HNK function as co-treatment with exogenous Wnt1 interferes with HNK-mediated growth inhibition whereas ICG-001 potentiates HNK efficacy. *In vivo* analyses of tumor xenografts from leptin-treated mice and obese-mice (physiologically hyperleptinemic) provide further evidence of the involvement of miR-34a, Wnt1-MTA1-β-catenin axes. Based on these data, we propose a model depicting a series of events including a feed-forward interaction of pStat3 and miR-34a, where pStat3 acts as a repressor of miR-34a, and effect of miR-34a on Wnt1-MTA1-β-catenin axis leading to its inhibition, which is important in HNK function.

Our studies offer the first evidence of the ability of HNK to activate miR-34a. This is an important finding as miR-34a has been reported as a tumor suppressor directly regulating various genes involved in diverse cellular processes, including cell proliferation, migration, invasion and EMT in many cancer types including breast cancer [43]. Recent studies have also shown that CD44 is a direct and functional target of miR-34a and enforced expression of miR-34a inhibits cancer stem cells, tumor regeneration and metastasis in prostate, osteosarcoma and renal carcinoma [44-46]. Our *in silico* analysis supports a previous finding showing that 3′untranslated region of Wnt1 is a functional target of miR-34a in dendritic cell differentiation [47]. Our previous studies have shown that Wnt1 regulates MTA1-β-catenin axis in breast cancer cells [15]. Here, we show that enforced miR-34a upregulation indeed potentiates HNK-mediated inhibition of Wnt1-MTA1-β-catenin axis while miR-34a inhibitor abrogates effect of HNK on Wnt1-MTA1-β-catenin axis. miRNAs are known to form negative or positive feedback loops with the transcription factors they directly or indirectly target allowing them to regulate multiple biological functions in response to initiating signals. The miR-34a also displays significant induction by tumor suppressor p53 whereas it is repressed by Snail which itself is downregulated via miR-34a [43]. We show a role of Stat3 in HNK-mediated activation of miR-34a where inhibition of repressive effects of Stat3 brings about miR-34a induction. Given the wide tumor-suppression activity of miR-34a, several concepts including nanovector-miRNA based delivery strategies of miRNA mimics have been put forth for systemic delivery of miR-34a. Though attractive, some of the concerns associated with nanovectors are low payload, complexity of the physical state of lipid carriers, and physicochemical stability. In light of these issues, a bioactive strategy such as HNK which is effective as well as non-toxic will have tremendous translational potential as an activator of miR-34a in cancer cells.

In conclusion, we uncover a novel role of HNK as an effective leptin-antagonist that inhibits leptin-induced growth of breast cancer cells *in vitro* and *in vivo*, which involves activation of miR-34a facilitating HNK-mediated inhibition of Wnt1-MTA1-β-catenin axis. We demonstrate a negative regulatory role of Stat3 participating in HNK-mediated activation of miR-34a whereby release of Stat3 from miR-34a promoter lifts the repressive effects of Stat3. Our results thus demonstrate the integral role of a previously unrecognized functional crosstalk between HNK and Stat3-miR-34a and Wnt1-MTA1-β-catenin axes. Considering the rising obesity pandemic and the fact that ~100,000 cancer-related deaths/year are attributed to obesity, our study demonstrating the efficacy of HNK towards breast cancer in obese state has important clinical implications and far-reaching impact. Although our studies are focused on breast cancer, we anticipate that the findings may apply broadly to other types of cancers. Knowledge gained from these studies lays foundation for future clinical studies investigating potential of HNK in preventing breast cancer progression in obese state and advances the field in a new direction.

## MATERIALS AND METHODS

### Ethics statement

The investigation was conducted in accordance with the ethical standards and guidelines approved by the authors' institutional review board (Johns Hopkins University IACUC).

### Cell culture and reagents

The human breast cancer cell lines, MCF7, and MDA-MB-231 were obtained from the American Type Culture Collection (ATCC, Manassas, VA), resuscitated from early passage liquid nitrogen vapor stocks as needed and cultured according to supplier's instructions. Cells were cultured for less than 3 months before reinitiating cultures and were routinely inspected microscopically for stable phenotype. Cells were treated with 100 ng/ml human recombinant leptin (Sigma, St. Louis, MO). We extracted Honokiol (HNK) from seed cone of *Magnolia grandiflora* according to our previously published study [48]. Antibodies for MTA1, β-catenin, cyclin D1, Stat3, phosphorylated-Stat3 and β-Actin were purchased from Cell Signaling Technology (Danvers, MA) and Santa Cruz Biotechnology, Inc. (Santa Cruz, CA). Stattic, Wnt1, and ICG001 were purchased from Sigma. Synthetic miRNA mimic and siRNA were purchased from Applied Biosystems (Ambion, Austin, TX).

### Clonogenicity and anchorage-independent and cell-viability assays

To perform clonogenicity assay [13], breast cancer cells were treated with 100 ng/ml leptin and 5μM HNK alone or in combination as indicated for 10-days; colonies were counted. Anchorage-independent growth of breast cancer cells in the presence of leptin and/or HNK was assayed by colony formation in soft agar [27]. Cell viability assay was performed using a commercially available XTT assay kit (Roche Applied Science, Indianapolis, IN).

### Invasion and spheroid migration assay

For an *in vitro* model system for metastasis, a Matrigel invasion assay [31] was performed by using a Matrigel invasion chamber from BD Biocoat Cellware (San Jose, CA). The slides were coded to prevent counting bias, and the number of invaded cells on representative sections of each membrane were counted under light microscope. The number of cells invaded Matrigel for each experimental sample represents the average of triplicate wells. Spheroids were formed following our previous protocol [28] and treated with leptin and/or HNK as indicated. After 48 hours of incubation, spheroids were fixed with 10% buffered formalin in PBS and stained with crystal violet. The migration of cells from spheroids was observed under light microscope.

### Breast tumorigenesis assay

MDA-MB-231 cells xenografts were generated; as previously described [27], grouped in four experimental groups. Mice were treated with intraperitoneal (IP) injections of 1) control (saline and Intralipid); 2) HNK, at 3 mg/mouse/day in 20% Intralipid (Baxter Healthcare, Deerfield, IL), three times per week; 3) recombinant leptin (dosage of 5 mg/kg), 5 days a week; 4) leptin and HNK for 4 weeks. The dose and route of HNK and leptin administration was selected from our previous studies documenting *in vivo* efficacy of honokiol and leptin [19, 27]. Tumors were regularly measured; collected after 4 weeks of treatment, weighed, and subjected to further analysis by immunohistochemistry, RT-PCR or western blot. At least four random, non-overlapping representative images from each tumor section from eight tumors of each group were captured using ImagePro software for quantitation of PCNA, MTA1, Wnt1, Cyclin D1, and β-catenin expression. Obese-mice model-Athymic nude mice were grouped in two groups-1) high-fat diet (HFD) and 2) normal diet (ND). HFD group were provided high-fat diet (Harlan: trans-fat custom diet TD110201, Harlan, Frederick, MD) and ND group were provided normal diet (Harlan: TD110196, designed as a control to HFD-TD110201, Harlan, Frederick, MD) (Table 1). Food consumption was monitored daily and body weights were monitored weekly. Blood samples were obtained and leptin levels measured using a mouse ELISA kit (ID Labs, Cambridge, MA, USA). HFD-mice exhibited considerable increase in body weight and leptin in 24 weeks. MDA-MB-231 cells xenografts were generated as previously described [27] after 24 weeks on HFD/ND administration. HFD and ND were maintained throughout the experiment. Mice were further grouped in experimental groups; 1) HFD + vehicle (saline and Intralipid); 2) HFD + HNK [3 mg/mouse/day in 20% Intralipid (Baxter Healthcare), oral gavage, three times per week]; 3) HFD + HNK [6 mg/mouse/day]; 4) ND + vehicle; 5) ND + HNK [3 mg/mouse/day]; 6) ND + HNK [6 mg/mouse/day]. Tumors were analyzed as discussed above. All animal studies were in accordance with the guidelines of Johns Hopkins University IACUC.

### Phospho-antibody array analysis

Breast cancer cells were treated with 5 μM HNK and the phospho-antibody array analysis was performed using the Proteome Profiler Human Phospho-Kinase Array Kit ARY003 from R&D Systems (Minneapolis, MN) according to the manufacturer's instructions. Array images were analyzed using the GeneTools image analysis software (Syngene, Frederick MD).

### Immunoblotting, RNA interference, immunofluorescence and confocal imaging

Cellular lysates were prepared following previously published protocol [49]. Immunoblotting was carried out as described [14]. The blots are representative of multiple independent experiments. *For RNA interference*, cells were transfected at 50% confluency with 100 nM of control siRNA or Stat3 siRNA (SignalSilence, Danvers, MA) using Oligofectamine (Cell Signaling Technology, Danvers, MA). Breast cancer cells were subjected to immunofluorescence analysis as described [27].

### Chromatin immunoprecipitation (ChIP) and RNA isolation, RT-PCR

ChIP analyses were performed using our published procedure [50]. For RNA isolation and RT-PCR, total cellular RNA was extracted using the TRIzol Reagent kit (Life Technologies, Inc., Rockville, MD). RT-PCR was performed using specific sense and antisense PCR primers. For qRT-PCR detection of miR-34a, miRNA-specific RT-primers (assay IDs: hsa-miR-34a, 000426), TaqMan miRNA Assay (Applied Biosystems, Ambion, Austin, TX) and Platinum Taq Polymerase Reagents (Invitrogen, Grand Island, NY) were used.

### Statistical analysis

All experiments were performed thrice in triplicates. Statistical analysis was performed using Microsoft Excel software. Significant differences were analyzed using student's *t* test and two-tailed distribution. Results were considered to be statistically significant if *p* < 0.05. Results were expressed as mean ± SE between triplicate experiments performed thrice.

## SUPPLEMENTAl MATERIAL FIGURES AND TABLE


