# Supplementary material for: Honokiol abrogates leptin-induced tumor progression by inhibiting Wnt1-MTA1-β-catenin signaling axis in a microRNA-34a dependent manner
Source: Oncotarget. 2015 Apr 15;6(18):16396–410. doi: 10.18632/oncotarget.3844 (PMC4599277; doi:10.18632/oncotarget.3844)
Supplement: Supplementary file 1 [file oncotarget-06-16396-s001.pdf]

# Honokiol abrogates leptin-induced tumor progression by inhibiting Wnt1-MTA1-β-catenin signaling axis in a microRNA-34a dependent manner

**Supplementary Material**

**Supplemental Data**

**Supplementary Methods**

***Clonogenicity assay*** To perform colony formation assay, breast cancer cells (single-cell suspension) were plated in 12-well plates at a density of 250 cells per well overnight. The following day, cells were treated with leptin and/or HNK and the medium was replaced with fresh medium containing treatments every 3 days. After a 10-day treatment period, the medium was removed and colonies were stained with crystal violet (0.1% in 20% methanol). Colony numbers were assessed visually and colonies containing >50 normal-appearing cells were counted. Pictures were taken using a digital camera.

***Anchorage-independent growth assay*** Anchorage-independent growth of breast cancer cells was assayed by colony formation in soft agar. Briefly, equal volumes of agar (1.2%) and complete medium were mixed to make 0.6% agar growth medium solution in 6-well tissue culture plates. Cells ($2x10^3$ cells/well) were suspended in media with or without leptin and/or HNK treatment followed by mixing with equal volume of agarose (0.6%). Cell suspension-agarose mix (2 ml) was then added to each well. Plates were incubated at 37ºC with 5% $CO_2$ in a humidified incubator for 3 weeks, and media with or without treatment were added every 3 days. Colonies were stained with 0.005% crystal violet in PBS for 1 hour at room temperature and observed using Olympus IX50 inverted microscope. Colonies were counted in five randomly selected fields at 10x magnification. Results are expressed as an average number of colonies counted per microfield.

***Cell viability assay*** Cell viability assay was performed by estimating the reduction of XTT (2, 3-bis(2-methoxy-4-nitro-5-sulfophenyl)-2H-tetrazolium-5-carboxyanilide), using a commercially available kit (Roche Applied Science, Indianapolis, IN) following manufacturer's instructions. Breast cancer cells were plated in 96 well plates at an initial density of $4 X 10^3$ cells / well for 24 hours followed by leptin and/or HNK treatment as indicated. XTT labeling reagent was added to each culture well to attain a final concentration of 0.3mg/ml.

After 4 hour exposure at $37^0$C, absorbance was measured between 450 and 690nm using a 96 well plate reader (SPECTRAmax PLUS, Molecular Devices, CA). Pilot experiments verified that the cell densities used in experiments performed were within the linear range of the XTT assay. A standard curve was prepared using cell densities from 1 X $10^3$ to 1 X $10^6$, and the results were calculated with respect to the number of cells.

***Phospho-Antibody Array Analysis*** The phospho-antibody array analysis was performed using the Proteome Profiler Human Phospho-Kinase Array Kit ARY003 from R&D Systems according to the manufacturer's instructions. Breast cancer cells were treated with HNK and lysed with Lysis Buffer 6 (R&D Systems). Preblocked nitrocellulose membranes of the Human Phospho-Kinase Arrays were incubated with 300 μg of cellular extracts overnight at 4 °C on a rocking platform, washed three times with 1x Wash Buffer (R&D Systems) followed by incubation with a mixture of biotinylated detection antibodies and streptavidin-HRP antibodies. Chemiluminescent detection reagents were used to detect spot densities. Array images were analyzed using the GeneTools image analysis software (Syngene). Every spot was subtracted by the averaged background level from negative control spots and normalized by the density levels of its own positive control spots. The averaged density of duplicated spots representing each phosphorylated kinase protein was determined and used for calculating the relative changes in phosphorylated kinase proteins. The list of target capture antibodies is available at http://www.rndsystems.com/pdf/ARY003.pdf.

***Immunofluorescence and confocal imaging*** Breast cancer cells ($5x10^5$ cells/well) subjected to immunofluorescence analysis. Fixed and immunofluorescently stained cells were imaged using a Zeiss LSM510 Meta (Zeiss) laser scanning confocal system configured to a Zeiss Axioplan 2 upright microscope with a 63XO (NA 1.4) plan-apochromat objective. All experiments were performed multiple times using independent biological replicates.

***Chromatin immunoprecipitation (ChIP)*** Chromatin samples were sonicated on ice three times for 10 seconds each (i.e., until the average length of sheared genomic DNA was 1 to 1.5 kb) followed by centrifugation for 10 minutes. The immunoprecipitated DNA was ethanol precipitated and re-suspended in 25 μl of water. Total input samples were re-suspended in 100 μl of water and diluted 1:100 before PCR analysis. Initially, PCR was performed with different numbers of cycles and/or dilutions of input DNA to determine the linear range of

amplification; all results shown fall within this range. Following 28-30 cycles of amplification, PCR products were run on 1% agarose gel and analyzed by ethidium bromide staining.

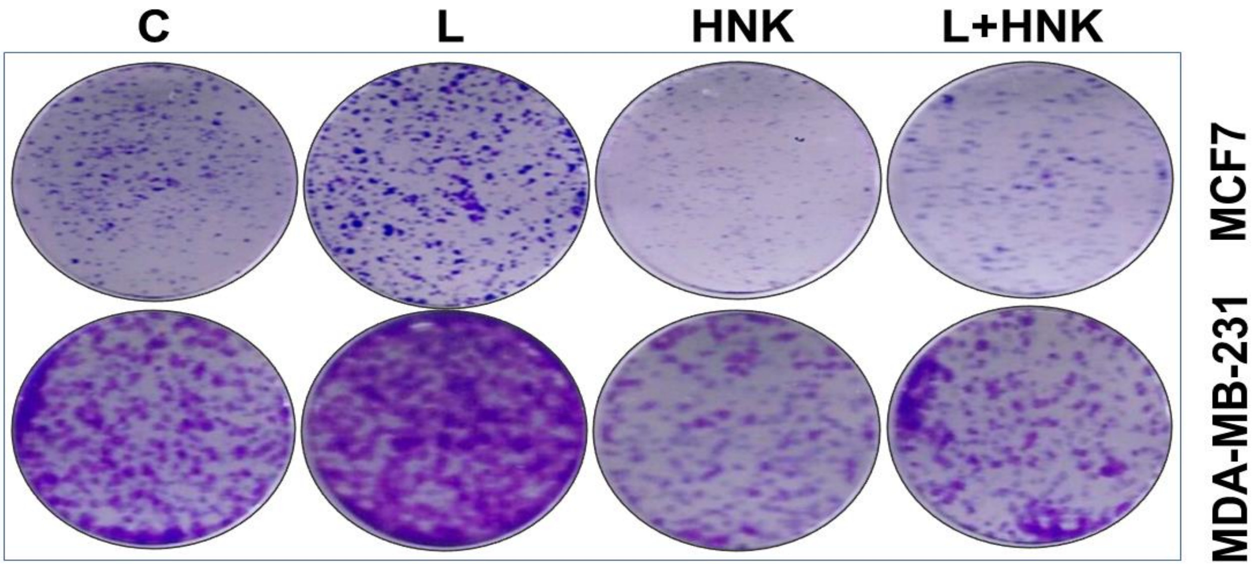


**Supplementary Figure 1: HNK inhibits leptin-induced clonogenicity of breast cancer cells.** Clonogenicity of breast cancer cells treated with 5 µM Honokiol (HNK) and 100ng/ml Leptin (L) (as indicated). Vehicle-treated cells are denoted with C.

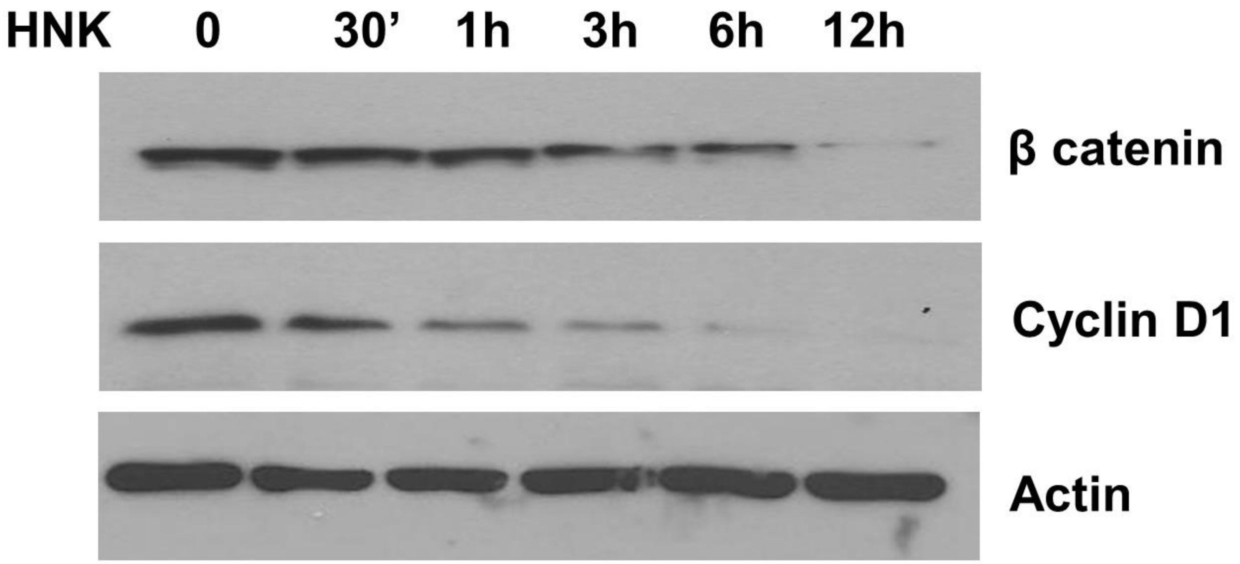


**Supplementary Figure 2: HNK treatment decreases the expression of β-catenin and cyclin D1.** MCF7 cells were treated with 5 µM HNK for various time intervals as indicated. Protein lysates were subjected to immunoblot analysis using β-catenin and cyclin D1 antibodies as indicated.

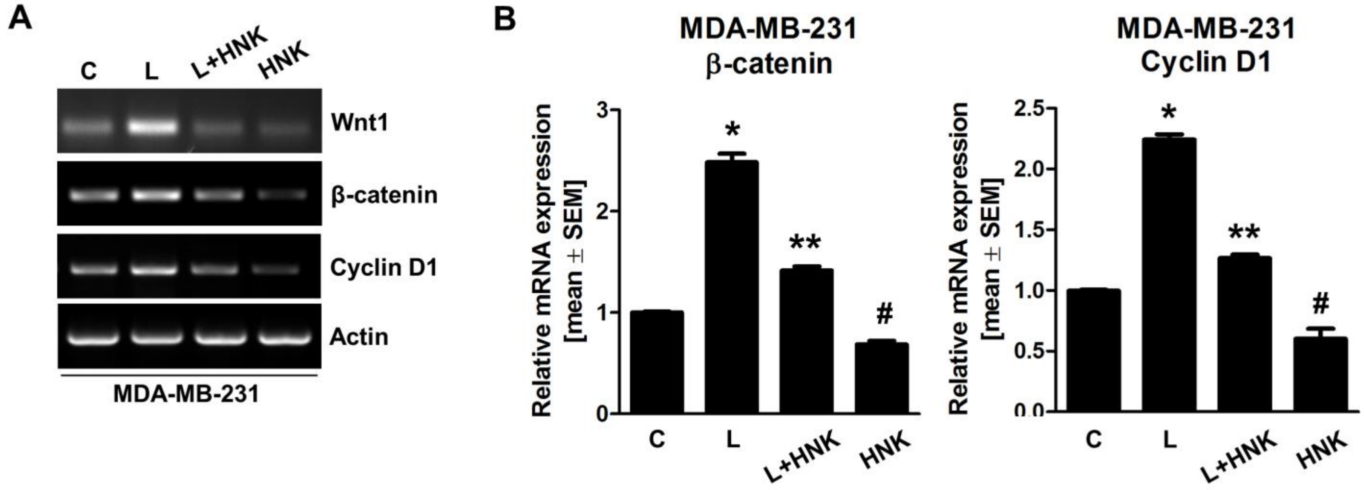


**Supplementary Figure 3: HNK decreases leptin-induced expression Wnt1, β-catenin and cyclin D1. (A)** RT-PCR analysis of Wnt1, β-catenin and cyclin D1 in breast cancer cells treated with vehicle (C), 5 μM HNK and 100ng/ml leptin (L) alone and in combination as indicated. **(B)** Real-time PCR analysis of β-catenin and cyclin D1 in breast cancer cells treated with vehicle (C), 5 μM HNK and 100ng/ml leptin (L) alone and in combination as indicated.

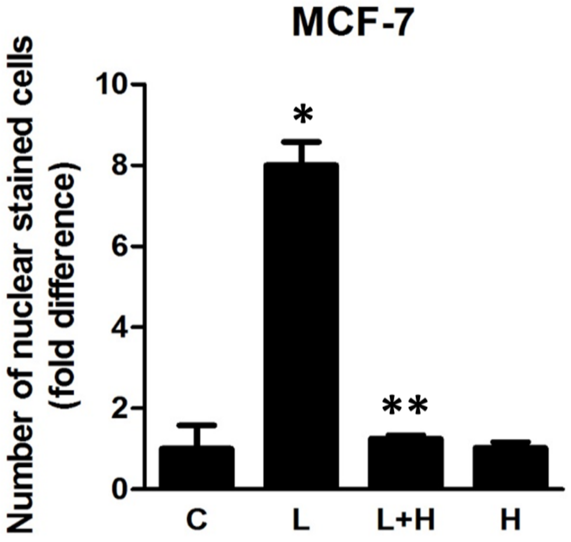


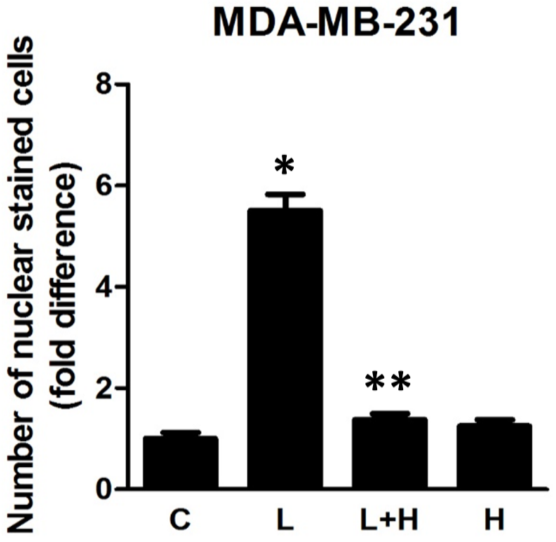


**Supplementary Figure 4: HNK decreases leptin-induced nuclear translocation of β-catenin.** Breast cancer cells were treated with 5 μM HNK and 100ng/ml leptin alone and in combination as indicated and subjected to immunofluorescence analysis of β-catenin. Nuclei were visualized with DAPI staining. Vehicle-treated cells are denoted with 'C'. Number of cells exhibiting nuclear translocation of β-catenin were counted and shown as bar graphs. *, $P<0.005$, compared with untreated controls, **, $P<0.005$, compared with leptin treated cells.

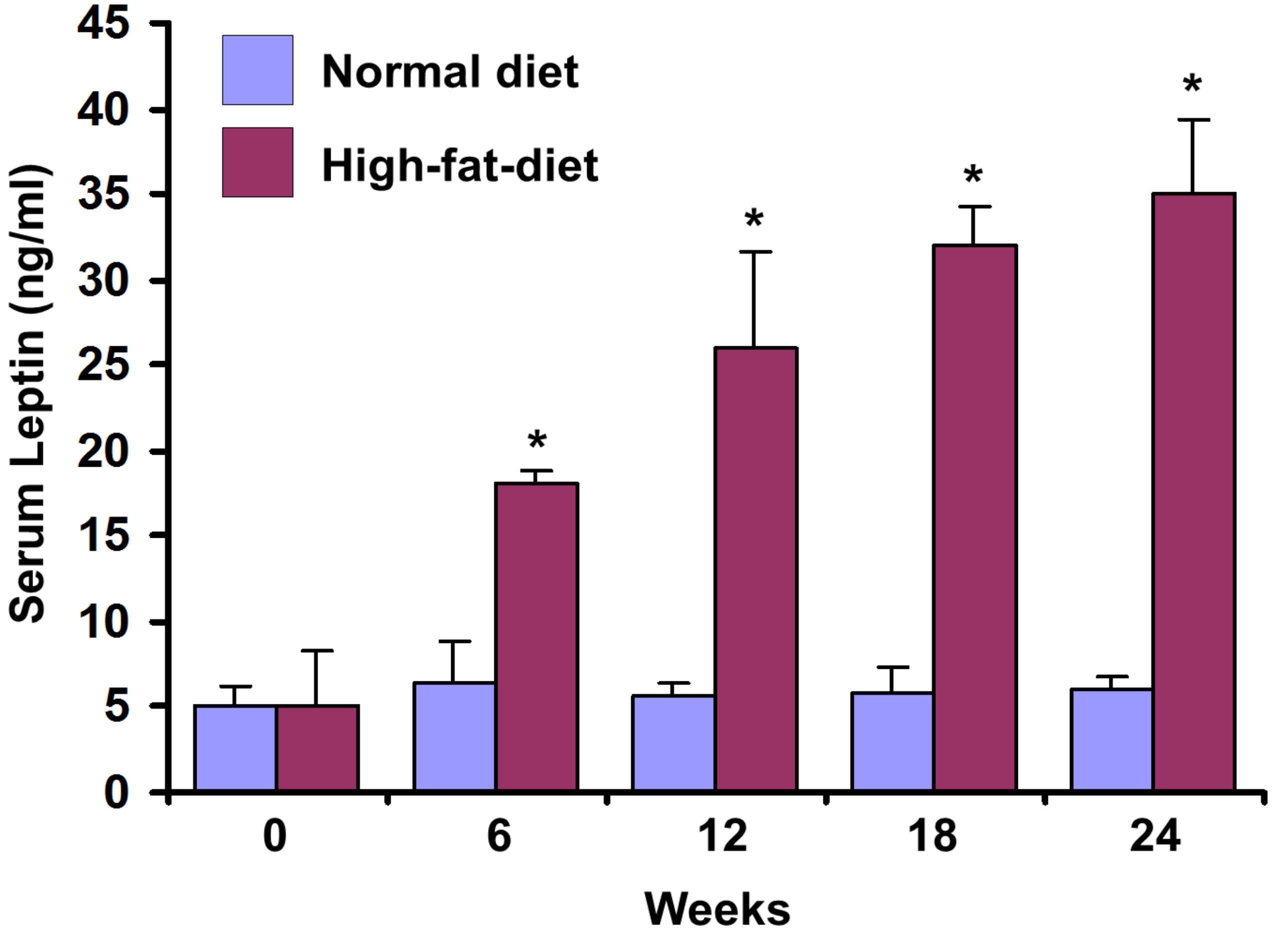


**Supplementary Figure 5: High-fat diet induces leptin levels.** Serum from mice after 6, 12, 24 weeks of high-fat diet was subjected to ELISA to quantify leptin levels. *, P<0.001, compared with untreated controls.

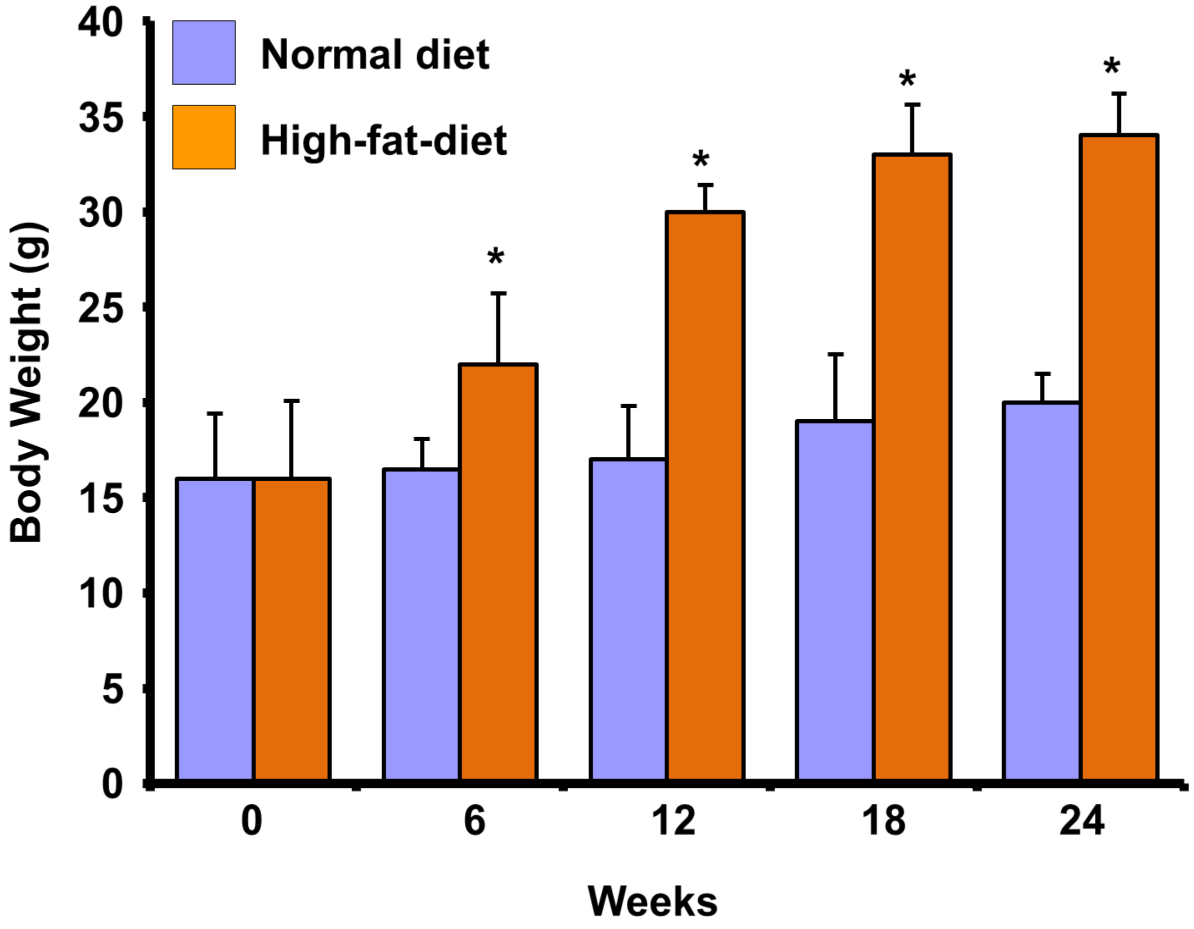


**Supplementary Figure 6: High-fat diet induces body weight gain** Body weights of mice after 6, 12, 24 weeks of high-fat diet were measured. (n=15). *, P<0.001, compared with untreated controls.

**Supplementary Table 1**

| **Formula and Nutrient Information of Research Diets** | | |
|---|---|---|
| **Group** | **HFD** | **ND** |
| **Diet*** | **TD110201 (g/kg)** | **TD110196 (g/kg)** |
| Casein | 230.0 | 183.0 |
| DL-Methionine | 3.4 | 2.7 |
| Sucrose, fine ground | 212.9277 | 100.0 |
| Corn Starch | 80.0 | 431.2751 |
| Maltodextrin | 140.0 | 130.0 |
| Vegetable Shortening, hydrogenated (Primex) | 220.0 | - |
| Soybean Oil | 10.0 | 60.0 |
| Cellulose | 50.0 | 50.0 |
| Mineral Mix, AIN-93G-MX (94046) | 46.0 | 36.8 |
| Calcium Phosphate, dibasic | 3.3 | 2.72 |
| Niacin | 0.063 | 0.051 |
| Calcium Pantothenate | 0.0336 | 0.027 |
| Pyridoxine HCl | 0.0147 | 0.012 |
| Thiamin HCl | 0.0126 | 0.0105 |
| Riboflavin | 0.0126 | 0.0105 |
| Folic Acid | 0.0042 | 0.0033 |
| Biotin | 0.0005 | 0.0003 |
| Vitamin B12 (0.1% in mannitol) | 0.0525 | 0.042 |
| Vitamin E, DL-alpha tocopheryl acetate (500 IU/g) | 0.15 | 0.15 |
| Vitamin A Palmitate (500,000 IU/g) | 0.0168 | 0.0135 |
| Vitamin D3, cholecalciferol (500,000 IU/g) | 0.0042 | 0.0033 |
| Vitamin K1, phylloquinone | 0.0016 | 0.0015 |
| Choline Bitartrate | 3.96 | 3.17 |
| TBHQ, antioxidant | 0.046 | 0.01 |

| **Selected Nutrient Information** | | | | |
|---|---|---|---|---|
| | **TD110201** | | **TD110196** | |
| | **% by weight** | **% kcal from** | **% by weight** | **% kcal from** |
| **Protein** | 20.4 | 17.7 | 16.2 | 17.6 |
| **Carbohydrate** | 42.8 | 37.1 | 62.0 | 67.3 |
| **Fat** | 23.2 | 45.2 | 6.2 | 15.1 |
| **Kcal/g** | 4.6 | | 3.7 | |

TD110196 is designed as a control for TD.110201 without trans-fat and with reduced sucrose. Protein, minerals and vitamins are adjusted so that they are equivalent to levels in TD.110201 when compared on the basis of kcal density.

*All diets were made by Harlan Tekland
